# The Role of Patients' Treatment Expectations in Ventricular Assist Device Support: Results From a Convergent Mixed Methods Study

**DOI:** 10.1111/aor.70175

**Published:** 2026-05-27

**Authors:** Simon Felix Zerth, Bernd Niemann, Miriam Salzmann‐Djufri, Sandra Semmig‐Könze, Katharina Tigges‐Limmer, Yvonne Brocks, Monika Sadlonova, Anna Markser, Tanja Zimmermann, Lara Kristin Oldach, Stefan Salzmann

**Affiliations:** ^1^ Division of Clinical Psychology and Psychotherapy, Department of Psychology Marburg University Marburg Germany; ^2^ Department of Cardiac‐, Thoracic‐ and Vascular Surgery University Medical Centre Goettingen Goettingen Germany; ^3^ Department of Cardiac and Vascular Surgery University Hospital Giessen Giessen Germany; ^4^ Department of Cardiac Surgery, Leipzig Heart Centre University of Leipzig Leipzig Germany; ^5^ Clinic for Thoracic and Cardiovascular Surgery, Heart and Diabetes Centre NRW University Clinic of the Ruhr University Bochum Bad Oeynhausen Germany; ^6^ Department of Psychosomatic Medicine and Psychotherapy University Medical Centre Goettingen Goettingen Germany; ^7^ Department of Geriatrics University Medical Centre Goettingen Goettingen Germany; ^8^ German Centre for Cardiovascular Research (DZHK) Partner Site Lower Saxony Goettingen Germany; ^9^ Clinical Institute for Psychosomatic Medicine and Psychotherapy University Hospital Duesseldorf Duesseldorf Germany; ^10^ Department of Psychosomatic Medicine and Psychotherapy, LVR‐Hospital Duesseldorf Hospital of the Heinrich Heine University Duesseldorf Duesseldorf Germany; ^11^ Department of Psychosomatic Medicine and Psychotherapy Hannover Medical School Hannover Germany; ^12^ Medical Psychology Health and Medical University Erfurt Germany; ^13^ Division of Clinical Psychology and Psychotherapy Vinzenz Pallotti University Vallendar Germany

**Keywords:** expectation violation, heart failure, LVAD, treatment expectations, VAD

## Abstract

**Objectives:**

Treatment expectations influence patient‐reported outcomes in chronic conditions; however, their role in ventricular assist device (VAD) therapy remains poorly understood. This study aimed to: (i) identify treatment expectations held by individuals with a VAD, (ii) assess the extent to which these expectations have been met, and (iii) examine how treatment expectations relate to VAD‐specific health‐related quality of life (HRQoL).

**Methods:**

A cross‐sectional convergent mixed methods design with equal weighting of the qualitative and quantitative parts was employed. Quantitative questionnaire data (*N* = 47) from adult individuals with a VAD were analyzed using descriptive statistics and Bayesian multiple linear regression. Qualitative data (*N* = 8) were obtained through semi‐structured interviews and analyzed using reflexive thematic analysis.

**Results:**

Participants reported having had high positive treatment expectations and moderate negative treatment expectations, on average. Approximately half of the participants indicated that they felt better than expected, while one‐third felt worse than expected. Bayesian multiple linear regression analysis revealed strong evidence for several expectation domains and expectation violation as relevant for HRQoL. The thematic analysis resulted in three themes: “No choice: survival as context for expectations,” “Shared experience and social support as mechanisms for coping and expectation adjustment,” and “Setting expectations through provider communication.”

**Conclusions:**

This mixed methods study suggests that treatment expectations play a significant role in VAD therapy and may be associated with HRQoL. Assessing and, in some cases, managing treatment expectations may improve VAD care and enable more informed decision‐making.

## Graphical abstract

This mixed methods study aimed to: (i) identify treatment expectations held by individuals with a ventricular assist device (VAD), (ii) assess the extent to which these expectations have been met, and (iii) examine how treatment expectations relate to VAD‐specific health‐related quality of life (HRQoL)

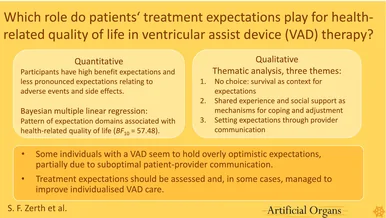

## 1 Introduction

Ventricular assist device (VAD) implantation represents a promising, life‐sustaining therapy for individuals with advanced heart failure [1]. As the number of donor hearts remains insufficient to meet current transplantation needs [2], VAD implantation has risen markedly in recent years [3]. While implantation typically leads to improvements in physical functioning [4], gains in psychological well‐being and health‐related quality of life (HRQoL) are less consistent. This variability reflects the complex process of adapting to life with mechanical circulatory support [5, 6].

Beyond medical factors, psychological factors likely influence post‐implantation HRQoL and disability [7, 8]. One such factor may relate to patients' treatment expectations, which represent a central mechanism of the placebo effect and seem to play a role in cardiac and surgical populations [9, 10, 11]. In the context of VAD therapy, patients' treatment expectations might relate to the expected benefit of a VAD, the experience of adverse events and side effects, or survival. While there is substantial evidence that more positive treatment expectations are linked to improved outcomes [9, 11], some studies suggest that overly optimistic expectations may be associated with poorer patient‐reported outcomes [12, 13].

To date, the relationship between treatment expectations and patient‐reported outcomes in individuals with a VAD has scarcely been investigated, and little is known about how unmet expectations might be associated with HRQoL. Qualitative findings indicate that patients might hold overly optimistic expectations which do not align with post‐implantation experiences [8, 14], leading to a violation of these expectations. Yet, a better understanding of patients' expectations is warranted.

Therefore, the present study aims to (i) identify pre‐implantation treatment expectations held by individuals with a VAD and to examine how these expectations are formed, (ii) assess the extent to which these expectations have been fulfilled or violated, and (iii) examine how treatment expectations and expectation violations are associated with VAD‐specific HRQoL.

A mixed methods approach was employed to capture both the subjective meaning and measurable association between treatment expectations and VAD‐specific HRQoL. Based on prior research, we hypothesized that more positive treatment expectations would be associated with better HRQoL. We further hypothesized that a negative mismatch between treatment expectation and lived experience would be associated with lower HRQoL.

## 2 Methods

### 2.1 Design

A cross‐sectional mixed methods design was employed. Qualitative data collected via telephone‐conducted semi‐structured interviews (approximately 30 min in duration) and quantitative data collected via validated questionnaires were gathered from February 2024 to May 2025 and analyzed convergently. This design enabled methodological triangulation by integrating qualitative and quantitative data to provide a more comprehensive understanding of VAD recipients' treatment expectations and HRQoL. The study adhered to the principles of the revised Declaration of Helsinki, was approved by the Gießen university medical center IRB (AZ188/23), and was pre‐registered on the Open Science Framework (https://doi.org/10.17605/OSF.IO/375FX).

### 2.2 Recruitment and Participant Inclusion

Eligible participants were individuals aged ≥ 18 years living with a VAD, capable of giving informed consent, and with sufficient German language proficiency. Individuals with a VAD were approached by staff psychologists or VAD coordinators of cooperating cardiac surgery departments, who provided flyers with study information and both a QR code and link to the survey platform for participation. The study flyer was additionally sent to VAD support groups and VAD‐specific online forums. Potential participants were informed about the study in writing and could then proceed to give their consent to participate, after which participants were asked to complete the questionnaires. The qualitative subsample represented a convenience sample of survey respondents who consented to further participation. Participants were not reimbursed. An a priori power analysis using G*Power (v. 3.1.9.6.) indicated a required sample size of *N* = 100 for a small‐to‐medium effect of *f* ^2^ = 0.10 via multiple linear regression with *α* = 0.05 and 1−*β* = 0.90. Recruitment was terminated after 14 months after exceeding the preregistered recruitment window and reaching the limit of personnel resources.

### 2.3 Data Collection

The following demographic data were collected: age, gender, education level, occupation status, marital status, months since VAD implantation, indication for VAD implantation.

Participants' recalled treatment expectations were assessed using the Treatment Expectation Questionnaire (TEX‐Q) [15], which measures patients' treatment expectations across various treatments multidimensionally on an 11‐point Likert scale. The TEX‐Q comprises six subscales measuring benefit, positive impact, negative impact, adverse events, process, and behavioral control expectations, respectively. The total score and subscales demonstrate good internal consistency (*α* = 0.71–0.92) and divergent validity with regard to measures of generalized expectations (*r* < 0.32) [15].

To assess the degree of violated expectations regarding (a) general health status and (b) side effect and adverse events, two items were created by the study team. Participants rated the extent to which their pre‐implant expectations differed from their current condition on an 11‐point numerical rating scale from 0 (“not at all”) to 10 (“completely”), and to indicate whether they were feeling better than expected, worse than expected, or exactly as they expected. The degree of expectation (mis‐)match could thus range from −10 (most negative expectation violation) to 10 (most positive expectation violation). A value of 0 indicated that participants felt exactly as they expected.

Ventricular assist device‐specific HRQoL was assessed using an adapted German version [16] of the Quality of Life with a Ventricular Assist Device (QoLVAD, matching US‐version 1) [17]. It evaluates five key domains of HRQoL in individuals with a VAD [18]: physical, emotional, social, cognitive, and meaning/spiritual. Items are rated on a 5‐point Likert scale (0–4) with a total score, ranging from 0 to 100, with higher scores indicating better HRQoL. The German version has recently been validated [16], showing excellent internal consistency for the total scale (*α* = 0.93) as well as convergent validity.

Moreover, depressive symptoms (Patient Health Quesionnaire‐9 [PHQ‐9]) [19], anxiety (Generalized Anxiety Disorder Scale‐7 [GAD‐7]) [20], optimism (Life‐Orientation‐Test—Revised [LOT‐R]) [21], and level of social support (ENRICHD Social Support Inventory [ESSI‐D]) [22] were assessed.

A semi‐structured interview guide (see Supporting Information S1) was developed by the lead investigator and reviewed by members of the study team. All interviews were conducted via telephone by the lead investigator, audio‐recorded, and manually transcribed verbatim.

### 2.4 Data Analysis

#### 2.4.1 Quantitative

Descriptive statistics were calculated for the TEX‐Q treatment expectation subscales, the total score, and the expectation violation measures. To identify which components of treatment expectations were most likely associated with VAD‐specific HRQoL, we employed Bayesian multiple linear regression with variable selection [23]. This Bayesian approach was chosen over traditional frequentist methods due to its ability to offer a more nuanced interpretation of uncertainty and evidence. This approach enables direct probabilistic inference about the strength of associations between variables, especially when sample size is small [24].

All analyses were conducted with JASP (version 0.95.4.0 [25]) using the software's default weakly informative prior for the regression coefficients and a neutral Beta‐Binomial (1,1) prior over model sizes. We report Bayes factors *BF*_10_, which quantify the relative evidence for the hypothesis that treatment expectations play a role in VAD‐specific HRQoL compared to the null hypothesis [26]. *BF*_10_ 3–10 can be interpreted as substantial evidence, *BF*_10_ 10–30 as strong evidence, and *BF*_10_ = 30–100 as very strong evidence [27].

#### 2.4.2 Qualitative

We performed reflexive thematic analysis (TA) [28] with the aim of developing themes, reflecting shared meaning within participants' accounts. Thematic analysis was conducted from an experiential perspective, deductive in nature, using the integrated model of expectations in patients undergoing medical treatment [10] as a framework through which we semantically coded and interpreted interview data. Two authors independently went through the first five phases of TA outlined by Braun & Clarke [29], that is, familiarizing with the data, generating initial codes, searching for and reviewing themes, defining and naming themes. While consensus coding was not aimed for, both coders regularly went over the data and codes, reviewing themes, enabling a more sophisticated understanding of the data. For coding, a TA macro for Microsoft Windows was used [30]. Selected quotes from the interviews were translated into English for reporting purposes.

## 3 Results

A total of *N* = 87 individuals accessed the study link. Of these, *n* = 8 declined to participate. Missingness was examined using Little's MCAR test [31], which indicated that the missingness pattern likely is completely at random (MCAR; *χ* ^2^ (708) = 620.33, *p* = 0.992). A case‐wise deletion approach was applied, excluding participants with insufficient data for key analyses. Thus, 17 individuals were excluded from the analysis as they consented to participate but did not enter any data. Another *n* = 7 participants were excluded since they only provided sociodemographic data, but did not fill out any questionnaires. After that, *n* = 8 participants were excluded due to not filling out primary questionnaires (TEX‐Q and/or QoLVAD). A total sample of *N* = 47 remained. When at least 80% of the items within a subscale were completed, missing responses were imputed using the respondent's mean score for that subscale. Eleven participants declared an interest to participate in the qualitative part of the study, all of which were contacted for interview purposes. Three participants could not be reached; all other participants were interviewed (*n* = 8), likely sufficient for meaningful theme development in this context [32].

### 3.1 Quantitative

#### 3.1.1 Sample Characteristics

Forty‐seven participants with a VAD were analyzed (mean age *M* = 56.85 ± 12.82 years, range: 24–74, 72.3% male). The majority of participants were partnered (*n* = 33; 71.22%) with a mean relationship duration of 28.31 years (±15.48). Most participants indicated having received a VAD as a bridge to transplant (*n* = 18; 38.30%), followed by destination therapy indication (*n* = 15; 31.91%). The mean time since implantation was approximately 2.58 years. Almost half of the sample reported moderate symptoms of depression [33], overall mild levels of anxiety [20], and a high (yet typical) degree of social support [22, 34]. Sample characteristics are presented in Table 1. The qualitative subsample included individuals with different treatment indications (bridge to decision *n* = 1, bridge to transplant *n* = 2, destination therapy *n* = 5).

**TABLE 1:** Sample characteristics.

Variable	Sample (*N* = 47)
Age (y)^a^	56.85 (±12.82)
Gender male, *n* (%)	34 (72.34)
Education secondary school diploma, *n* (%)	17 (36.17)
Occupation pension, *n* (%)	32 (68.10)
Marital status partnered, *n* (%)	33 (71.22)
Length of relationship (y)^a^	28.31 (±15.48)
Time since VAD implantation (m)^a^	30.89 (±28.99) (Mdn = 23.50) Range (1; 155)
Treatment Indication, *n* (%)	
Bridge to transplant (BTT)	18 (38.30)
Bridge to recovery (BTR)	4 (8.51)
Bridge to decision (BTD)	4 (8.51)
Destination therapy (DT)	15 (31.91)
Unknown to participant	6 (12.77)
Health‐related quality of life (QoLVAD)	60.18 (±19.08)
Treatment expectations (TEX‐Q)	6.38 (±1.15)
Depression (PHQ‐9)	9.11 (±5.82)
PHQ‐9 ≥ 10	20 (45.5)
Anxiety (GAD‐7)	5.84 (±4.98)
GAD‐7 ≥ 10	9 (20.5)
Optimism (LOT‐R)	10.39 (±5.82)
Social support (ESSI‐D)	21.14 (±3.81)

*Note:* PHQ‐9 total score ≥ 10 is a proposed screening cut‐off score for detecting depression [33, 35]; GAD‐7 total score ≥ 10 is a proposed cut‐off score for moderate symptoms of anxiety [20]; ESSI total score < 18 is a proposed cut‐off score for low perceived social support [34].

Abbreviation: Mdn, median.

^a^ values are presented as means (± standard deviation); y, years; m, months.

#### 3.1.2 Treatment Expectations and Expectation Violation

**FIGURE 1:**
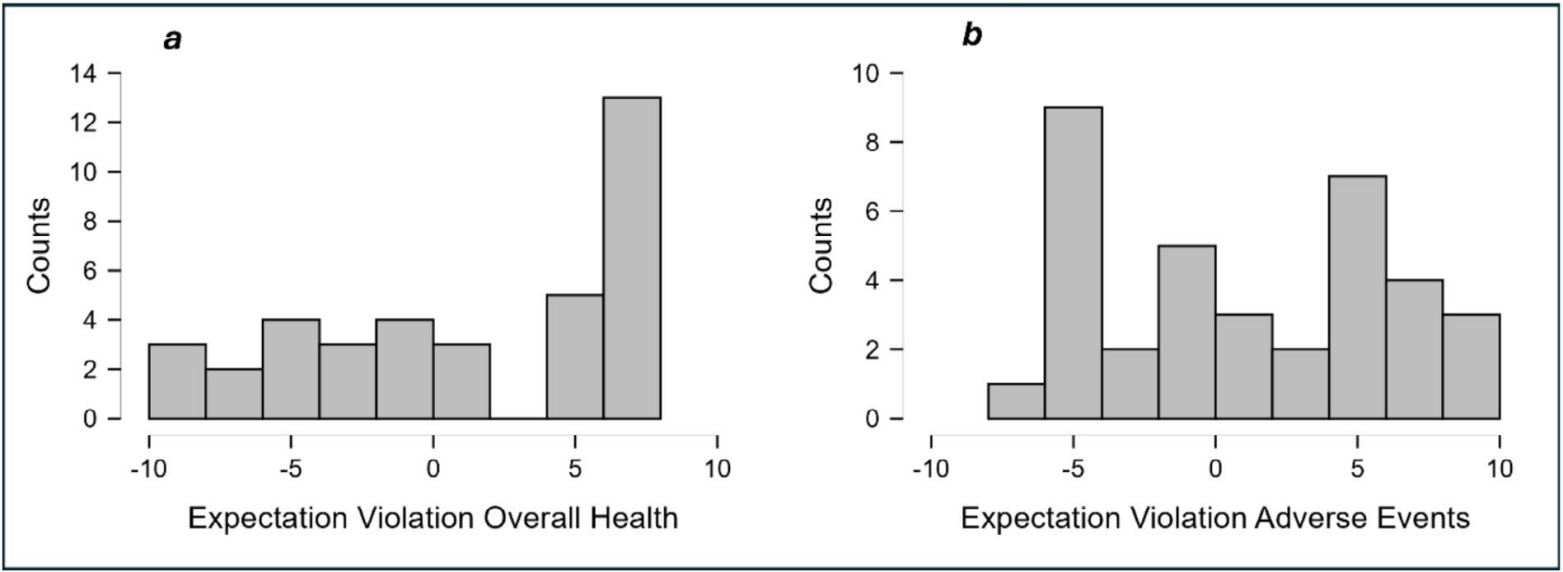
Frequency distribution of reported expectation violations. Panel a shows expectation violations regarding participants' overall health status. Panel b shows expectation violations regarding experienced adverse events. Zero indicates a match between expectation and experience, negative values represent negative expectation violations (feeling worse than expected), positive values represent positive expectation violations (feeling better than expected) [Color figure can be viewed at wileyonlinelibrary.com].

Participants indicated pronounced benefit (*M* = 7.27, SD = 2.11) and positive impact expectations (*M* = 7.21, SD = 2.10). Expectations regarding adverse events (*M* = 5.55, SD = 2.24) and a negative impact of VAD treatment (*M* = 5.11, SD = 2.62) were lower on average. Participants indicated moderate process expectations (*M* = 6.44, SD = 2.42) and pronounced behavioral control expectations (*M* = 7.91, SD = 2.08). Regarding the (mis‐)match of expectations and post‐implantation experience for their overall health status, 56.75% of participants reported that they felt better than expected. About one third of participants (32.43%) reported that they felt worse than expected (see Table 2). Visual inspection indicates a unimodal distribution with a pronounced peak at the positive end of the expectation violation continuum (see Figure 1a). In contrast, the frequency distribution of expectation violations regarding adverse events and side effects indicates a bimodal distribution with two peaks at either midpoint of the positive and negative valence, respectively (see Figure 1b). For side effects and adverse events, analogously to the overall expectation violation, a similar pattern emerged with the majority of participants feeling better than expected (52.78%) and a third of participants feeling worse than expected (33.33%).

**TABLE 2:** Treatment expectations and expectation violations reported by the sample.

Variable	*M* (SD)
Benefit expectations (TEX‐Q, [0;10])	7.27 (2.11)
Positive impact expectations (TEX‐Q, [0;10])	7.21 (2.10)
Adverse events expectations (TEX‐Q, [0;10])	5.55 (2.24)
Negative impact expectations (TEX‐Q, [0;10])	5.11 (2.62)
Process expectations (TEX‐Q, [0;10])	6.44 (2.42)
Behavioral control expectations (TEX‐Q, [0;10])	7.91 (2.08)
Total score (TEX‐Q, [0;10])	6.38 (1.14)
Expectation violation regarding overall health [−10;10]	1.57 (5.78)
*n*, positive expectation violation (%)	21 (56.75)
*n*, negative expectation violation (%)	12 (32.43)
*n*, no expectation violation (%)	4 (10.81)
Expectation violation regarding adverse events and side effects [−10;10]	1.39 (5.15)
*n*, positive expectation violation (%)	19 (52.78)
*n*, negative expectation violation (%)	12 (33.33)
*n*, no expectation violation (%)	5 (13.89)

*Note:* Square brackets indicate range of scale.

Abbreviation: TEX‐Q, Treatment Expectation Questionnaire.

#### 3.1.3 Associations Between Treatment Expectations and VAD‐Specific HRQoL

Bayesian linear regression was conducted to examine the associations between expectation domains as well as expectation violation and VAD‐specific HRQoL. The model including expectations towards positive impact, adverse events, behavioral control, as well as expectation violation regarding participants' overall health was best supported by the data, showing very strong evidence relative to the null model (*BF*_10_ = 57.48).

Posterior summaries indicated that higher behavioral control expectations were positively associated with HRQoL (*M* = 2.58, SD = 2.08, 95% CI [−0.06, 6.68]). Expectations towards adverse events and side effects (*M* = −0.97, SD = 2.08, 95% CI [−5.29, 3.71]) and, surprisingly, positive impact expectations (*M* = −2.70, SD = 2.61, 95% CI [−8.04, 0.88]) were negatively associated with HRQoL. A positive expectation violation regarding the overall health status (feeling better than expected) was positively associated with HRQoL (*M* = 0.71, SD = 0.78, 95% CI [−0.11, 2.47]). Inclusion Bayes factors for these variables ranged from *BF*_inclusion_ = 1.32 to 3.28, suggesting anecdotal to moderate evidence for individual predictors (see Supporting Information S1).

Overall, although the combined model substantially outperformed the null model, the uncertainty in individual parameter estimates suggests that a joint pattern of expectations, rather than specific domains, may be most relevant for explaining differences in HRQoL.

### 3.2 Qualitative

Three themes were developed, “No choice: survival as context for expectations,” “Shared experience and social support as mechanisms for coping and expectation adjustment,” and “Setting expectations through provider communication.” These three conceptually distinct themes map onto different levels of influence, namely intrapersonal and interpersonal (including medical providers). Further, “expectation violations” representing the degree of (mis‐)match between expectation and lived reality were developed as an overarching process, which relates to these themes independently.

#### 3.2.1 No Choice: Survival as Context for Expectations

Participants frequently stated that they felt that they had no choice other than to accept receiving a VAD, as the other option would have meant death.

They told me that the system was necessary and so on and so forth, and I couldn't get used to the idea at all. […] They said it was going downhill fast and, ‘you probably won't live to see your birthday’. (Participant 1)

I spent six months sedated, lying around in some hospital beds, um, let me put it this way, I wasn't aware of what awaited me. The only question I asked myself back then was: Do I want to keep living, or do I want this to be the end? (Participant 8)

Participants recounted that, due to this threatening context, they did not develop overly elaborated expectations, but rather assumed that VAD therapy reliably worked as a life‐sustaining measure and that they had a similar quality of life than before.

You don't have another choice, another option. You rely on it, assume that everything will be fine, that you will, let's say, maintain a certain quality of life. (Participant 4)

You know, I didn't really have this one specific… how should I put it… expectation beforehand. I said to myself, okay, if this keeps me alive, that's good, very good. (Participant 1)

The central organizing concept was that, when confronted with this existential situation, participants drew on fundamental aspects of their personality or worldview to make sense of the experience, and to navigate it emotionally. Most prominently, participants described optimism, which can be understood as a generalized outcome expectation [10], and stoicism as key internal resources. Depending on the degree of such traits within a participant, this was described as helpful, provided guidance, while also shaping expectations participants held about treatment and recovery.

I had the feeling that it's just not my time yet […]. So I went into the surgery with great optimism, also with the awareness or confidence that I wasn't the first patient the team had done this with. (Participant 2)

To be completely honest, I didn't have specific expectations at all. I just let myself go with the flow, I'm a Stoic, as they say. I grew up with this philosophy, and approaching everything with serenity is the non plus ultra. So I was completely calm when I went into surgery. (Participant 6)

Drawing on internal resources such as optimism appeared to have helped participants regain a sense of control and agency within an otherwise externally determined, life‐threatening situation. Similarly, participants seemed to enhance their perceived agency by developing behavioral control or outcome expectations; specifically, the belief that their own actions could meaningfully influence the process of rehabilitation and adjusting to life with a VAD.

Recovery was hard work, of course. It was a struggle, definitely. With rehab and everything. But, um, I also expected that I would have to contribute a lot myself. (Participant 7)

Although approaching implantation with optimism was described helpful by many, some participants found that the realities of life after implantation did not match their pre‐implantation expectations or desired state, especially in the face of complications.

I was informed, but I approached the matter with optimism, of course. Unfortunately, it didn't turn out as positively as I had hoped. (Participant 7)

#### 3.2.2 Shared Experience and Social Support as Mechanisms for Coping and Expectation Adjustment

In addition to participants' traits and personal philosophies, external social factors also played a significant role in how participants navigated the existential situation, formulated expectations, and ultimately experienced their quality of life. In particular, support from spouses and partners, as well as contact with other VAD recipients, was described as especially influential.

The other thing, I kind of got myself out of that one and said, ‘yeah, I don't feel able to… decide that for myself. I'd like to talk to my wife and the rest of my family about it first…And [discuss] what can be done and so on’. (Participant 1)

And the expectation was actually… I just let it happen. Beforehand I… well, my wife and I didn't know what an LVAD was – one, two, three weeks before the operation. So we googled it, or rather my wife googled it and then briefly explained it to me – and then she told me it was the only option, and I said, okay. (Participant 4)

Throughout participants' accounts, peer support from other VAD recipients played a meaningful role. For individuals who felt overwhelmed by their new reality, connecting with peers who were further along in the process provided reassurance, practical insight, and a form of understanding that they felt was often lacking among medical professionals outside of specialized VAD centers.

I continued to look for psychological support from home, but I have to say that I didn't have a very good experience at first. That's because we're simply exotic. Many people are afraid of us. (Participant 3)

Well, I have a few people – I always call them my little community – whom I talk to on the phone quite often, both men and women, who also have an LVAD system. And I'm kind of, um, we're talking about five people here, and I'm currently the tough case when it comes to the whole infection situation. (Participant 7)

And when you engage with each other within a group like that, you don't need to explain much. They know how you're doing and they know that there are good days and bad days and you just learn how to deal with it. And that's a really big relief. (Participant 3)

Conversely, participants who described themselves as coping relatively well described that acting as a peer supporter gave them a sense of agency, reinforcing their own adjustment while contributing to others' journeys.

And then the doctor asked me if I would go to the ward and talk to a patient who was unsure whether to go ahead with it or not. I was happy to do that, so I went to the ward and talked to him for almost an hour, explaining my own situation and so on. And yes, I hope he made a positive decision. (Participant 2)

I spoke to [another VAD patient], went to his room; the physician noticed and then moved him to my room a day or two later, and we talked. The young man said that without me, he wouldn't have pulled through […]. And then I formed a group, we meet every four weeks. It's important to talk to each other. (Participant 4)

#### 3.2.3 Setting Expectations Through Provider Communication

The influence of medical providers' communication on expectation formation was a common theme throughout, also with regard to how participants reported feeling post‐implantation, presumably at least partially mediated through a (mis‐)match between expectation and post‐implantation experience. Central for this theme was the idea that, since VAD implantation often can be regarded as a VAD‐or‐death situation, it was in the medical professionals' best interest that participants agreed to receiving a VAD. According to VAD recipients' accounts, this was achieved through communicating positive outcome expectations unilaterally and leaving out information on possible side effects or adverse events. Participants emphasized that, in most cases, they felt that they were taken care of adequately by their doctors. However, in some cases, participants felt as if doctors were “selling” the VAD to them, particularly when the information received did not seem credible and was overly positive. Expectation violations mostly concerned burdens that participants weren't aware of, often relating to the equipment and limitations that come with it as well as side effects.

The conversations always went like this… They said […] a woman flew to Australia to visit her children; and another went on a trip to the North Cape and went canoeing and paddling, everything went well and there were no difficulties. Then [I thought], hm, I'll actually be supplied by a device… with a pump, a normal pump, and then I'll have a controller outside my body for the power supply… That can't possibly work without any problems. (Participant 1)

Of course, they tell you that you can go on vacation or do whatever you want, like take a cruise. They also give you information about [the VAD] – but you know yourself that there are a lot of things to consider. […] I didn't know that I would later have to deal with equipment like batteries and control units, and that it would be such a handicap. (Participant 4)

In some instances, participants wished that they would have received more information, even if this had occurred post‐implantation.

I feel well advised and well treated. They gave me a controller and the relevant documentation, which I was able to read and familiarize myself with. The only thing would be […] that they say: here's a bag, hang it around your neck, you'll be walking around with it all the time. (Participant 2)

## 4 Discussion

This mixed methods study aimed to explore how treatment expectations and expectation violations are formed and experienced by individuals with a VAD, and to examine the extent to which these expectations are associated with VAD‐specific HRQoL.

Quantitatively, participants reported having had high “positive” treatment expectations (i.e., benefit, positive impact, process, and behavioral control expectations) and less pronounced “negative” treatment expectations (i.e., adverse events, and negative impact expectations). While approximately half of the sample indicated that they felt better than expected, around one third recounted that they felt worse than anticipated, both regarding their overall health and regarding side effects and adverse events. When these findings are considered together with the qualitative findings, it becomes apparent that participants' pre‐implant expectations often did not align with their subsequent experiences. Although many described being broadly satisfied with their overall health after receiving a VAD, they simultaneously reported notable negative expectation violations. In particular, participants recounted unanticipated burdens relating to living with the device. Given the only moderately expected burdens, these negative expectation violations are not surprising, particularly in the light of prior research documenting a wide range of challenges and complications associated with VAD implantation and post‐implant adjustment [6].

Bayesian multiple linear regression revealed a combination of expectation domains (i.e., behavioral control, side effect and adverse event expectations, positive impact expectations) as well as expectation violation regarding the participants' overall health status as potentially meaningful variables with very strong evidence. Posterior summaries indicated anecdotal to moderate evidence for individual variables, as well as large credible intervals, suggesting that treatment expectations may operate less as isolated domains and more as part of a broader expectation context. This interpretation is congruent with qualitative findings, where participants rarely described their expectations as singular, but rather as broad and intertwined.

Notably, positive impact expectations were negatively associated with HRQoL, which is unexpected, given that positive expectations are typically linked to better outcomes in chronic conditions (e.g., [9]). This finding becomes comprehensible when viewed together with participants' qualitative accounts, in which expectation mismatches were described repeatedly. Positive impact expectations, particularly those conveyed by healthcare professionals, were often described as unmet or overly optimistic, and some individuals expressed skepticism about these expectations. It is therefore plausible that individuals who internalized these highly positive expectations were later confronted with disappointment when their post‐implantation reality did not align with what had been communicated. This is in line with the few studies explicitly considering expectations in individuals with ventricular assist devices [8, 14].

### 4.1 Clinical Implications

Several implications can be drawn from these findings. First, treatment expectations and the degree to which they align with post‐implant experiences should be viewed as integral to the adjustment process and to HRQoL. Ventricular assist device recipients form expectations both independently and in interaction with their environment (family, VAD peers, medical providers). It is therefore essential that treatment expectations are assessed and discussed to ensure that care is optimally tailored to the individual [36]. Drawing on evidence from placebo research on patient‐provider communication, these conversations should occur in a warm and competent manner [37], with the latter including the formulation of positive yet realistic expectations. Recent work in cancer care indicates that managing patients' expectations is recognized as part of patient‐centered communication; clinicians attempt to foster positive treatment expectations, although they note that overly positive framing can undermine trust when the lived experience falls short [38]. While it is understandable that medical teams may wish to guide patients towards choosing VAD therapy (particularly when it represents the only life‐sustaining option), overly optimistic communication should be avoided for ethical reasons as well as to prevent substantial negative expectation violations, possibly affecting HRQoL and the adjustment process.

At the same time, clinicians must remain mindful of the nocebo risks associated with excessive or overly detailed disclosure of potential burdens [39]. Although a nocebo‐sensitive communication requires careful disclosure about potential burdens and side effects, participants' qualitative accounts suggested that some had not been informed about fundamental aspects of VAD therapy. Future research should investigate optimized informed consent communication strategies in the context of VAD implantation. Evidence from other fields suggests that expectation‐focused informed consent consultations can enhance patients' knowledge and strengthen their capacity to provide informed consent [40].

Our findings also suggest potential value in peer‐based support. Where desired, access to VAD peer groups may help individuals with a VAD cope and adapt, given that participants repeatedly reported feeling most understood by other VAD peers. This is in line with the finding that social support predicts improved patient‐reported outcomes in individuals with a VAD [5].

### 4.2 Limitations

This study has several strengths, such as the mixed methods design, the use of a multidimensional assessment of treatment expectations, and the VAD‐specific assessment of HRQoL. However, several limitations warrant consideration. First, the study design was cross‐sectional and both expectations and expectation violations were assessed retrospectively. Consequently, the findings must be interpreted as correlational, even though the qualitative data provide some insight into potential directional processes that the quantitative data cannot establish. Second, while a Bayesian approach was chosen for its robustness, statistical analyses were likely underpowered and the wide credible intervals observed for several predictors limit the precision of inference about the contribution of individual expectation domains. The findings therefore speak more to the overall pattern of expectations than to the relevance of specific domains. Third, the relatively small sample size limits generalizability and recruitment relied on individuals who were well enough to participate, which might have introduced bias. Larger‐scale trials with pre‐ and post‐implantation assessments, including information on the type of provider conducting patient education and its content, are warranted.

## 5 Conclusions

Mixed methods evidence suggests a pattern of expectation domains, likely influenced by patient‐provider interaction, which is associated with VAD‐specific HRQoL. Disappointed expectations may be preventable through expectation‐focused, placebo and nocebo‐sensitive communication, an employment of which might be beneficial for VAD recipients and should be investigated in future research.

## Author Contributions

**Simon Felix Zerth:** conceptualization, methodology, formal analysis, investigation, data curation, visualization, project administration, writing – original draft. **Bernd Niemann:** supervision, resources, writing – review and editing. **Miriam Salzmann‐Djufri:** resources. **Sandra Semmig‐Könze:** resources. **Katharina Tigges‐Limmer:** resources, writing – review and editing. **Yvonne Brocks:** resources, writing – review and editing. **Monika Sadlonova:** resources, writing – review and editing. **Anna Markser:** resources. **Tanja Zimmermann:** resources, writing – review and editing. **Lara Kristin Oldach:** formal analysis, writing – review and editing. **Stefan Salzmann:** conceptualization, project administration, supervision, writing – review and editing.

## Funding

The authors have nothing to report.

## Ethics Statement

All procedures performed in studies involving human participants were in accordance with the ethical standards of the institutional research committee and with the 1964 Helsinki declaration and its later amendments or comparable ethical standards.

## Consent

Informed consent was obtained from all individual participants included in the study.

## Conflicts of Interest

The authors declare no conflicts of interest.

## Supporting information


**Data S1:** aor70175‐sup‐0001‐Supinfo.docx.

## Data Availability

Quantitative data generated from publicly available questionnaires are available on request from the corresponding author. Qualitative data generated from interviews are not publicly available due to privacy and ethical restrictions.
