# Supplementary material for: The Role of Patients' Treatment Expectations in Ventricular Assist Device Support: Results From a Convergent Mixed Methods Study
Source: Artif Organs. 2026 May 27;50(9):1305–14. doi: 10.1111/aor.70175 (PMC13525227; doi:10.1111/aor.70175)
Supplement: Supplementary file 1 [file AOR-50-1305-s001.docx]

**Original interview guide (German)**

**Halbstrukturierter Interviewleitfaden**

*Vielen Dank, dass Sie sich zu einem Interview bereiterklären. Es bedeutet uns viel, Ihre persönliche Geschichte zu hören. Ihr Beitrag zu unserer Studie soll helfen, ein besseres Verständnis von psychologischen Aspekten zu bekommen, die für die Anpassung an ein Leben mit einem Herzunterstützungssystem eine Rolle spielen. Langfristig soll uns dies helfen, ein psychologisches Unterstützungsangebot zu entwickeln. Das Gespräch wird ungefähr eine halbe Stunde dauern.*

- Könnten Sie mir zu Beginn erzählen, wie lange Sie bereits ein Herzunterstützungssystem haben?
- Zuerst soll es nun um das Thema Erwartungen gehen. Die folgende Frage bezieht sich auf die Zeit, **bevor** Sie das Herzunterstützungssystem implantiert bekommen haben. Wenn Sie an die Zeit zurückdenken, als klar war, dass Sie ein mechanisches Herzunterstützungssystem bekommen werden - Was haben Sie von der Behandlung, von dem Herzunterstützungssystem, erwartet?
  - Haben Sie Positives von der Behandlung erwartet? Wenn ja, was?
  - Haben Sie Negatives von der Behandlung erwartet? Wenn ja, was?
  - *Prompts:*
    - Was haben Sie erwartet, inwieweit sich … verändern würde?
      - Lebensqualität
      - Körperliches Wohlbefinden (u.a. Symptome)
      - Psychisches Wohlbefinden
    - Wenn „nichts“ erwartet: Was hat Sie beschäftigt? Wovor hatten Sie Angst?
- Die folgende Frage bezieht sich nun auf die ersten Wochen und Monate, **nachdem** Sie Ihr Herzunterstützungssystem implantiert bekommen haben. Inwieweit wurden Ihre Erwartungen an das Leben mit dem mechanischen Herzunterstützungssystem erfüllt oder enttäuscht?
  - *Prompts*: Wieder auf vorher genannte Bereiche eingehen, falls auf diese nicht von selbst Bezug genommen wird
    - Lebensqualität
    - Körperliches Wohlbefinden (u.a. Symptome)
    - Psychisches Wohlbefinden
- Die folgende Frage bezieht sich auf Ihre **aktuellen** Erwartungen. Was erwarten Sie weiterhin für die Zukunft in Bezug auf das Leben mit Ihrem Herzunterstützungssystem?
  - *Prompts*: Wieder auf vorher genannte Bereiche eingehen, falls auf diese nicht von selbst Bezug genommen wird
    - Lebensqualität
    - Körperliches Wohlbefinden (u.a. Symptome)
    - Psychisches Wohlbefinden
- Haben sich Ihre **Erwartungen** an das VAD und Ihr Leben damit zu irgendeinem Zeitpunkt auf Ihr Wohlbefinden ausgewirkt? Beschreiben Sie bitte.
  - positive/negative/“keine“ Erwartungen vor der Implantation
  - Erfüllte/ „Verletzte“ Erwartungen
- Wie hätte man Sie im Hinblick auf das Thema Erwartungen an das Herzunterstützungssystem, vor oder nach der Implantation, unterstützen können? Wie hätte sich eine solche Unterstützung auf Sie und Ihr Umfeld ausgewirkt?
- Zum Thema **Erwartungen**: Gibt es noch etwas, das wir nicht gefragt haben, was Ihnen aber noch einfällt? Würden Sie gerne etwas ergänzen?
- Nun soll es um **psychologische Unterstützung** und Gesprächsangebote im weiteren Sinne gehen. Hätten Sie zu irgendeinem Zeitpunkt gerne psychologische Unterstützung in Anspruch genommen?
  - Zu welchem Zeitpunkt?
  - Was hätten Sie sich dadurch erhofft?
- [falls in Anspruch genommen, was hatten Sie sich erhofft und waren Sie damit zufrieden?]
- Wenn Sie die Möglichkeit gehabt hätten, ein unterstützendes Gespräch mit einem Psychologen zu führen, was wäre Ihnen dabei wichtig gewesen?
  - Was wären Themen, über die Sie bei einem solchen Gespräch gerne reden würden?
  - Was wären Ihre Erwartungen an ein einmaliges Gesprächsangebot?
  - *Prompt Partnerschaft*: Fänden Sie es wichtig, dass zukünftige Gesprächsangebote Ihre/n Partner/in miteinbinden?

**[Zum Ende kommen, nach noch offenen Fragen oder Ergänzungen fragen, sich Bedanken und Verabschieden]**

**Translated interview guide**

**Semi-structured interview guide**

*Thank you very much for agreeing to take part in this interview. It means a lot to us to hear your personal story. Your contribution to our study will help us gain a better understanding of the psychological aspects that play a role in adapting to life with a ventricular assist device. In the long term, this will help us develop psychological support services. The interview will take about half an hour.*

- To begin with, could you please tell me how long you have had a ventricular assist device?
- First, we would like to talk about expectations. The following question relates to the time **before** you had the ventricular assist device implanted. When you think back to the time when it became clear that you would be receiving a VAD, what did you expect from the treatment; from the VAD?
  - Did you expect anything “positive” from the treatment? If so, what?
  - Did you expect anything “negative” from the treatment? If so, what?
    - Prompts: To what extent did you expect … to change?
      - Quality of life
      - Physical well-being (including symptoms)
      - Psychological well-being
    - If expected “nothing”: What was on your mind? Was there anything you were afraid of?
- The following question relates to the first few weeks and months **after** you had your ventricular assist device system implanted. To what extent have your expectations of life with a VAD been met or disappointed?
  - Prompts: Refer back to the areas mentioned above if they are not referred to spontaneously.
- The following question related to your **current** expectations. What are your expectations for the future in terms of living with your VAD?
  - Prompts: Refer back to the areas mentioned above if they are not referred to spontaneously.
- Have your **expectations** of the VAD and your life with it affected your well-being at any point? Please describe.
  - Positive/negative/”no expectations” prior to implantation
  - Fulfilled/disappointed expectations
- How could you have been supported with regard to your expectations of living with a VAD, either before or after implantation? How would such support have affected you and those around you?
- On the subject of **expectations**: Is there anything else we haven’t asked you about that comes to mind? Would you like to add anything else?
- Now let us talk about psychological support and counselling in a broader sense. Would you have liked to have sought psychological support at any point?
  - At what point?
  - What would you have hoped to gain from it?
    - [If they did seek support: How did you hope to benefit from it and were you satisfied with it?]
- If you had had the opportunity to have a supportive conversation with a psychologist, what would have been important to you?
  - What topics would you have liked to discuss in such a conversation?
  - What would your expectations be for such a conversation?
  - Prompt relationship: Would you find it important for future conversation offers/interventions to involve your partner?

**[conclude by asking if there are any remaining questions or additions, express gratitude, goodbye to the participant]**

| ***Model Comparison – Total scale* QoLVAD** | | | | | |
| --- | --- | --- | --- | --- | --- |
| Models | P(M) | P(M\|data) | BF_M_ | BF_10_ | R² |
| Null model | 0.100 | 0.025 | 0.231 | 1.000 | 0.000 |
| TEXQ_mean + TEXQ_benefit + TEXQ_posimpact + TEXQ_adverse + TEXQ_negimpact + TEXQ_process + TEXQ_behavcontr + Expectation_Violation_overall + Expectation_Violation_adverse | 0.100 | 0.094 | 0.929 | 3.732 | 0.491 |
| TEXQ_mean + TEXQ_benefit + TEXQ_posimpact + TEXQ_adverse + TEXQ_negimpact + TEXQ_process + TEXQ_behavcontr + Expectation_Violation_overall | 0.011 | 0.020 | 1.783 | 7.052 | 0.487 |
| TEXQ_posimpact + TEXQ_behavcontr | 0.003 | 0.018 | 6.715 | 26.363 | 0.335 |
| TEXQ_mean + TEXQ_posimpact + Expectation_Violation_overall | 0.001 | 0.017 | 14.303 | 56.159 | 0.421 |
| TEXQ_behavcontr | 0.011 | 0.013 | 1.155 | 4.599 | 0.179 |
| TEXQ_posimpact + TEXQ_adverse + TEXQ_behavcontr + Expectation_Violation_overall | 7.937×10^-4^ | 0.011 | 14.567 | 57.483 | 0.466 |
| TEXQ_mean + TEXQ_benefit + TEXQ_posimpact + TEXQ_adverse + TEXQ_process + TEXQ_behavcontr + Expectation_Violation_overall + Expectation_Violation_adverse | 0.011 | 0.010 | 0.935 | 3.732 | 0.491 |
| TEXQ_mean + TEXQ_benefit + TEXQ_adverse + TEXQ_negimpact + TEXQ_process + TEXQ_behavcontr + Expectation_Violation_overall + Expectation_Violation_adverse | 0.011 | 0.010 | 0.935 | 3.732 | 0.491 |
| TEXQ_mean + TEXQ_benefit + TEXQ_posimpact + TEXQ_negimpact + TEXQ_process + TEXQ_behavcontr + Expectation_Violation_overall + Expectation_Violation_adverse | 0.011 | 0.010 | 0.935 | 3.732 | 0.491 |
|  | | | | | |
| *Note.*  Table displays only a subset of models; to see all models, select "No" under "Limit No. Models Shown". | | | | | |

**Posterior Summary**

| *Posterior Summaries of Coefficients* | | | | | | | | | |
| --- | --- | --- | --- | --- | --- | --- | --- | --- | --- |
|  | | | | | | | | 95% Credible Interval | |
| Coefficient | P(incl) | P(excl) | P(incl\|data) | P(excl\|data) | BF_inclusion_ | Mean | SD | Lower | Upper |
| Intercept | 1.000 | 0.000 | 1.000 | 0.000 | 1.000 | 59.600 | 2.869 | 53.732 | 64.946 |
| TEXQ_total | 0.500 | 0.500 | 0.592 | 0.408 | 1.451 | 2.291 | 7.306 | -10.571 | 18.080 |
| TEXQ_benefit | 0.500 | 0.500 | 0.463 | 0.537 | 0.861 | 0.098 | 1.935 | -4.262 | 4.867 |
| TEXQ_posimpact | 0.500 | 0.500 | 0.742 | 0.258 | 2.872 | -2.702 | 2.614 | -8.670 | 0.522 |
| TEXQ_adverse | 0.500 | 0.500 | 0.568 | 0.432 | 1.315 | -0.966 | 2.081 | -5.673 | 2.086 |
| TEXQ_negimpact | 0.500 | 0.500 | 0.510 | 0.490 | 1.043 | -0.520 | 1.576 | -4.157 | 2.043 |
| TEXQ_process | 0.500 | 0.500 | 0.469 | 0.531 | 0.884 | -0.297 | 1.229 | -2.847 | 2.516 |
| TEXQ_behavcontr | 0.500 | 0.500 | 0.766 | 0.234 | 3.276 | 2.582 | 2.082 | -0.016 | 6.480 |
| Expectation_Violation_overall | 0.500 | 0.500 | 0.659 | 0.341 | 1.931 | 0.706 | 0.782 | -0.290 | 2.392 |
| Expectation_Violation_adverse | 0.500 | 0.500 | 0.473 | 0.527 | 0.897 | -0.052 | 0.757 | -1.926 | 1.558 |
|  | | | | | | | | | |
